# Commentary: Case Report: Giant atypical granular cell tumor of the median nerve

**DOI:** 10.3389/fonc.2026.1825942

**Published:** 2026-07-21

**Authors:** Tomonori Kawasaki, Atsushi Enomoto, Masanori Wako, Naofumi Taniguchi, Jiro Ichikawa

**Affiliations:** 1 Department of Pathology, Saitama Medical University International Medical Center, Hidaka, Saitama, Japan; 2 Department of Pathology, Nagoya University Graduate School of Medicine, Nagoya, Aichi, Japan; 3 Department of Orthopaedic Surgery, Interdisciplinary Graduate School of Medicine, University of Yamanashi, Chuo, Yamanashi, Japan

**Keywords:** atypical, Fanburg-Smith classification, granular cell tumor, malignant, Nasser classification

## 1 Introduction

Granular cell tumors (GCTs) are neurogenic neoplasms that rarely arise in the extremities or deep soft tissues (1, 2). We read with interest the recent publication by Liu et al., titled “Case report: Giant atypical granular cell tumor of the median nerve” (3). In GCT diagnosis, distinguishing between benign, atypical, and malignant forms is important, as the prognosis, including recurrence and metastasis, varies. However, the diagnostic criteria and classification of GCTs remain controversial (4, 5).

## 2 Subsections relevant for the subject

We provide an overview of GCTs, discuss the histopathological findings, and highlight key points for distinguishing between benign, atypical, and malignant forms.

## 3 Discussion

### 3.1 Overview of GCTs

GCTs, originating from Schwann cells, can arise in various anatomical locations (6). However, they most commonly occur in the skin, subcutaneous tissues, and head and neck region (6). Deep-seated lesions in the extremities are rare (2). In contrast, cases involving peripheral nerves, such as the present case, are considered reasonable, given the neurogenic origin. In addition to the median nerve, GCTs arising from the ulnar (7) and posterior tibial (8) nerves have been reported. Magnetic resonance imaging (MRI) is crucial in GCT diagnosis (5). On T1-weighted images, the signal intensity is typically isointense relative to the muscle, whereas on T2-weighted images, it ranges from isointense to mildly hyperintense and often appears heterogeneous. Contrast-enhancement patterns vary (2, 9). Although characteristic MRI findings specific to intraneural GCT have not been well-documented, intramuscular GCTs reportedly exhibit distinctive features, such as a peripheral rim and stripe signs that represent continuous muscle fibers entrapped within the tumor (2, 9, 10). Although definitive imaging features to distinguish benign from malignant GCTs remain unclear, certain findings, such as heterogeneous high signal intensity on T2-weighted images, areas of necrosis, infiltration into surrounding tissues, and poorly defined margins, are potential indicators of malignancy (2, 9). FDG-PET shows promise in differentiating benign from malignant lesions, with malignant GCTs exhibiting higher SUVmax values. Further studies are required to validate these observations (11).

### 3.2 Histopathological findings and genetic features of GCTs

Characteristic histopathological features of GCTs include ill-defined borders and sheets and nests of large, epithelioid-to-polygonal cells with eosinophilic granular cytoplasm. In some cases, lymphocytic infiltration and lymphoid follicle formation occur (1, 5, 6). Immunohistochemical analyses play critical roles in the differential diagnosis of GCTs. Although positivity rates vary slightly among studies, commonly positive markers include S100, SOX10, inhibin, neuron-specific enolase, calretinin, and CD68 (5, 6). The differential diagnosis of GCTs is broad, encompassing benign and malignant neoplasms and inflammatory lesions (5, 6). Genetic alterations including monosomy 22, trisomy 10, and loss of distal 5p as well as *CDKN2A* have been reported (5). Mutational analysis identified PIK3CA and TP53 as key drivers in malignant GCT (5). Among malignant tumors, distinguishing GCTs from melanoma, malignant peripheral nerve sheath tumors, and leiomyosarcoma is important (5).

### 3.3 Key points in the differential diagnosis of benign, atypical, and malignant GCTs

**Figure 1:**
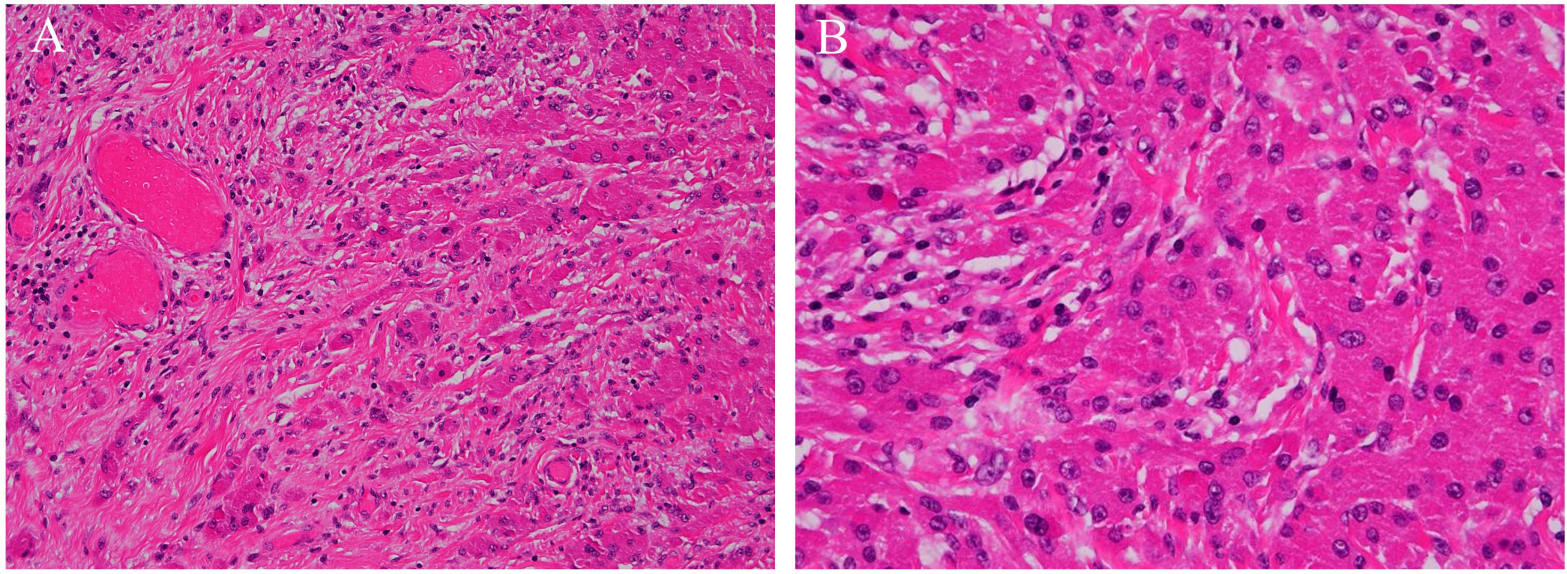
Histopathological findings of an atypical granular cell tumor. Spindling tumor cells [**(A)** ×200] and vesicular nuclei [**(B)** ×400] are shown. H&E stain.

GCTs are classified into benign, atypical, and malignant. Fanburg-Smith et al. proposed six histological criteria for this classification: necrosis, spindling, vesicular nuclei with large nucleoli, increased mitotic activity, high nuclear-to-cytoplasmic (N/C) ratios, and pleomorphism (**Table 1**) (4). We present the histopathological features of an atypical GCT (**Figure 1**). Tumors exhibiting none or only the focal presence of these features are considered benign; those with one or two features are classified as atypical; and those with three or more features are diagnosed as malignant (4). However, this system has several limitations. First, there is interobserver variability, particularly in the assessment of nuclear atypia and the N/C ratio, which are subjective and lack reproducibility (5, 12). Second, the correlation between classification and clinical prognosis is imperfect; for instance, rare cases diagnosed as benign using the Fanburg-Smith criteria have been reported to metastasize (5, 12). To address these issues, Nasser et al. proposed a simplified and reproducible diagnostic approach that focused on two key features: necrosis and increased mitotic activity (**Table 1**) (12). According to these criteria, tumors exhibiting either or both of these features are classified as GCTs with uncertain malignant potential (UMP), whereas those with confirmed metastases are diagnosed as malignant. In their study, cases deemed malignant according to the Fanburg-Smith criteria were reclassified as GCT-UMP and those previously labeled as atypical were considered benign (12). No reclassified benign cases experienced recurrence or metastasis (12). Conversely, Imanishi et al. reported detailed clinical outcomes of cases diagnosed as atypical or malignant using the Fanburg-Smith criteria (13). Two atypical GCT cases developed metastases and experienced tumor-related death. Both tumors were large, measuring 10 and 17 cm, respectively. The Ki-67 labeling index was 5% in atypical cases and 9.4% in malignant cases, suggesting a trend toward higher proliferative activity in malignant tumors. However, an atypical case with a low Ki-67 index of 1% also resulted in tumor-related death (13). Although the sample size was limited and the results warrant cautious interpretation, these findings suggest that even atypical GCTs with large tumor sizes may behave aggressively; such cases should be managed considering the possibility of malignancy. In the case reported by Liu et al., four Fanburg-Smith criteria were fulfilled, supporting the diagnosis of malignant GCT. According to Nasser’s classification, these tumors can be categorized as GCT-UMPs. However, considering the large tumor size and high Ki-67 index of 10%, we believe that a clinical diagnosis of malignant GCT is appropriate. Although the surgical margin status was not described in this report, this information is critical. As the surgical approach in the case of Liu et al. likely aimed to preserve the median nerve, margin assessment is particularly important. In the study by Imanishi et al., all cases of atypical or malignant GCTs with positive margins experienced recurrence; however, it is important to recognize that recurrence can occur even when surgical margins are negative (13). Moreover, recurrence and metastasis can occur more than 3 years postoperatively, underscoring the need for long-term follow-up (13). Considering these factors, a follow−up period of at least five years is warranted. Finally, although surgical excision remains the mainstay of therapy, owing to the infiltrative behavior of the tumor, recurrence may occur even after wide resection. Radiotherapy and chemotherapy have been used in cases of recurrence or metastasis; however, their efficacy remains uncertain (13).

**Table 1:** Fanburg-Smith’s classification and Nasser’s classification.

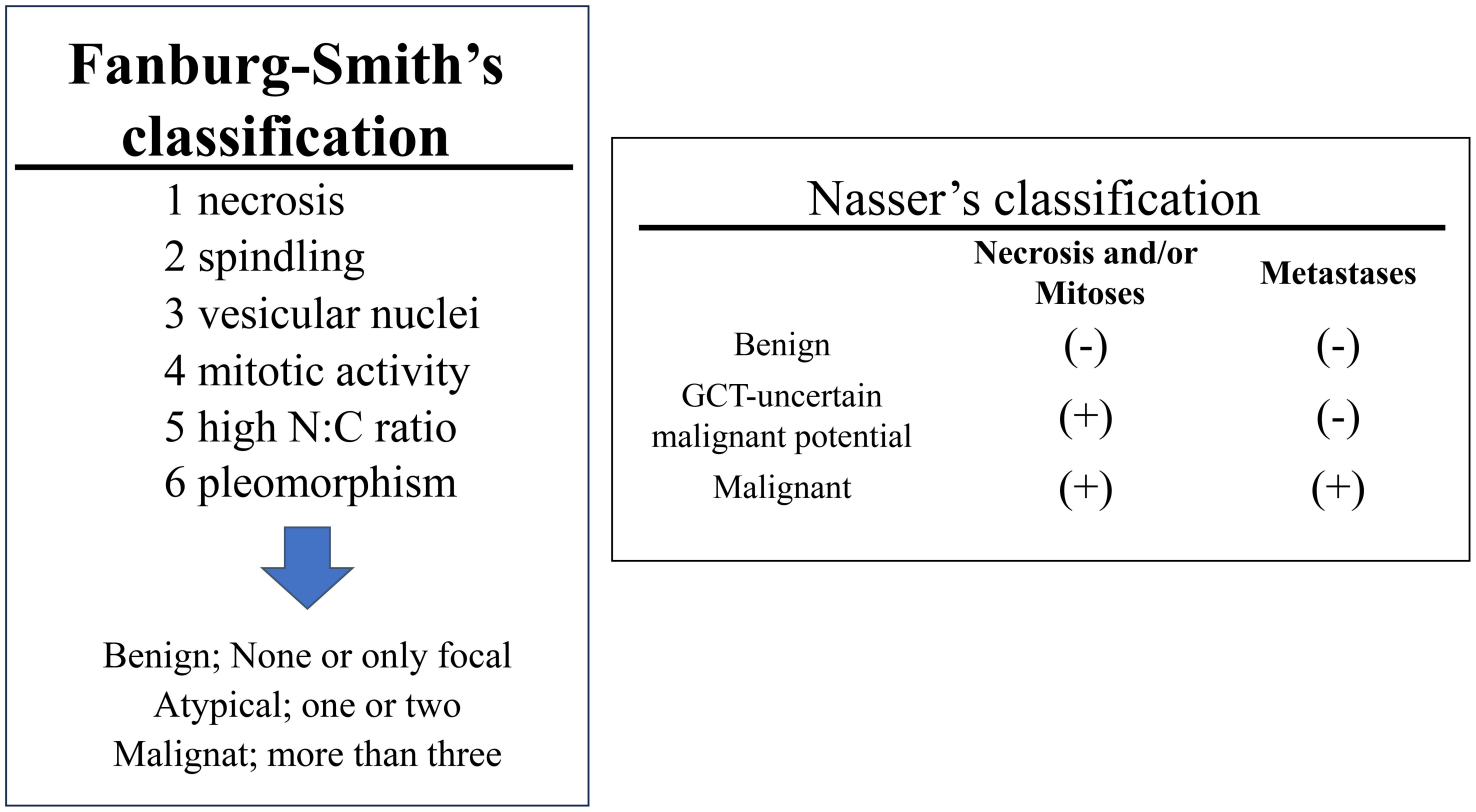

## 4 Conclusions

The current diagnostic criteria for benign, atypical, and malignant GCTs have yet to be defined. However, given the high recurrence and metastasis rates associated with malignant GCT, diagnostic accuracy must take precedence over convenience. Further comprehensive clinicopathological analyses involving a larger case series are warranted to establish more reliable classification standards.
